# Tobacco and alcohol use in Tunisian young doctors: a way to evade occupational stressors

**DOI:** 10.1192/j.eurpsy.2023.1393

**Published:** 2023-07-19

**Authors:** M. A. Ghrab, I. Sellami, A. Abbes, M. Hajjaji, K. Jmal Hammami, M. L. Masmoudi

**Affiliations:** 1 Occupational medicine, University Hospital Hedi Chaker, Sfax, Tunisia; 2 University of Sfax, Sfax, Tunisia

## Abstract

**Introduction:**

The medical field is very stressful. To reduce stress, healthcare workers may resort to different habits, including smoking and drinking.

**Objectives:**

We aimed to assess the smoking and drinking habits of interns and fellows in Tunisian hospitals.

**Methods:**

A cross-sectional study was conducted in April 2022 through online platforms. A pre-established questionnaire was sent to Tunisian medical interns and fellows, working in public hospitals, and collected sociodemographic and occupational data. The Fagerstörm test was used to evaluate nicotine dependence.

**Results:**

Our population consisted of 182 Tunisian interns and fellows. Their mean age was 26.38±2.03 years. Females represented 71.4% of the total population. One hundred of them (54.95%) were fellows, out of which 18% specialized in surgery. Sixty-one per cent of these young doctors were single. Twenty-two individuals were smokers with male predominance (59.1%). Smoking was associated with age (p<0.001) and female sex (p=0.001). Fagerstörm test score’s mean was 4.09±2.52. High to very high nicotine dependency was found in 31.8% of cases. Thirty-five interns and fellows consumed alcohol and 51.4% of them were females. Alcohol use was associated with sex (p<0.001).

**Conclusions:**

Despite knowing their hazard, young doctors still resort to drinking and smoking as a coping mechanism. The promotion of healthier coping mechanisms is essential.

**Disclosure of Interest:**

None Declared

